# Improving the Advantages of Single Port in Right Hemicolectomy: Analysis of the Results of Pure Transumbilical Approach with Intracorporeal Anastomosis

**DOI:** 10.1155/2012/874172

**Published:** 2012-04-10

**Authors:** Salvador Morales-Conde, Antonio Barranco, María Socas, Cristina Méndez, Isaias Alarcón, Jesús Cañete, Francisco J. Padillo

**Affiliations:** Unit of Innovation in Minimally Invasive Surgery, University Hospital “Virgen del Rocío”, 41010 Sevilla, Spain

## Abstract

*Background*. Single-port laparoscopic surgery has recently emerged as a method to improve patient recovery and cosmetic benefits of laparoscopic surgery. The evolution of our technique has led us to move from a periumbilical incision to a transumbilical one, avoiding the use of drain and maintaining a pure single-port approach with intracorporeal anastomosis in order to maintain the incision as smaller as possible. *Method*. We report a prospective clinical analysis of our first 38 patients. Oncological surgical steps were followed as during the standard laparoscopic approach, performing the anastomosis intracorporeally in all cases. *Results*. Mean age of 68,39 years old and an average BMI of 27,88%. (range 19,81–41,5). Most lesions were adenocarcinoma (65,8%), while the remaining were polyps (31,5%) and one a mucocele of the appendix. We moved from a periumbilical incision, initial 14 cases, into a transumbilical one, (medium size of the incision 3,25 cm). Average surgical time was 117,42 minutes. Drains was only used in our first 3 cases. Mean hospital stay was 5,2 days, (86,5% stayed less than 5 days). Total morbidity was 13%. Histological exams of the specimens showed that the oncological criteria were preserved. *Conclusions*. Single-port right hemicolectomy with intracorporeal anastomosis is feasible and safe. The advantages of a total intracorporeal anastomosis include that there is no need to enlarge the umbilical incision and avoid traction of the pedicle of the mesenterium of the transverse colon during the extracorporeal anastomosis. A transumbilical incision offers better cosmetic results, and the use of drains can be avoided, which increase, patient's satisfaction.

## 1. Introduction

Laparoscopic surgery for carcinoma of the colon is a feasible technique as short- and long-term results show. This technique is as safe and effective as the open approach [1, 2]. The development of minimally invasive surgical techniques tries to search for new methods and approaches to improve cosmetic results, reduce postoperative pain, and minimize possible complications associated to laparoscopic approach, trying at the same time to preserve the oncological results so far obtained with the standard laparoscopic procedures. New approaches, such as NOTES and single-port access surgery, are being developed in the field of minimally invasive surgery. In fact, single-port access surgery is becoming accepted in some laparoscopic procedures such as cholecystectomy [3, 4], nephrectomy [5], appendectomies [6], adrenalectomies [7], splenectomies [8], bariatric procedures [9], and colonic surgery [10]. Even that this approach has demonstrated to be feasible in colonic surgery, further efforts are necessary to prove if surgeons may obtain similar results, in terms of morbidity and oncological results, to those obtained by standard laparoscopic approach.

On the other hand, we have to keep analyzing our results in order to determine the best way of performing these procedures. There is still a great debate in order to determine where to place the single-port devices, the way of performing the incision in the umbilicus, transumbilical versus periumbilical, the instruments to be used, straight versus curve versus Roticulator instruments, and, in case of right colonic resections, how to perform the anastomosis, extracorporeal versus intracorporeal.

## 2. Patients and Methods

### 2.1. Case Series

We report a prospective clinical analysis of our first 38 pure single-port right colonic resection performed between June of 2009 and November of 2011. We analyse the evolution of our technique as well as the morbidity and the oncological results of our series.

### 2.2. Surgical Technique

**Figure 1:**
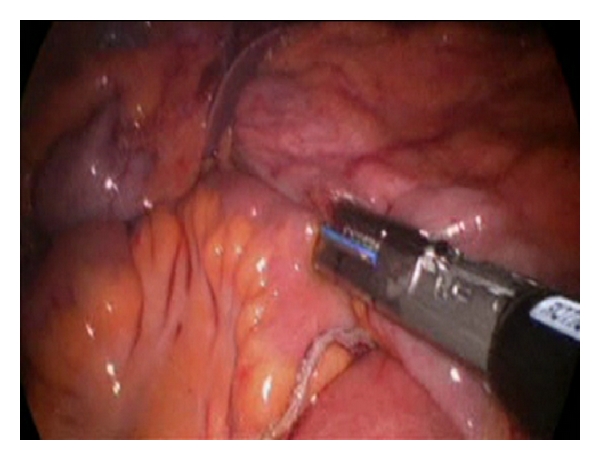
Intracorporeal anastomosis using an Endo Stapler with a blue cartridge.

**Figure 2:**
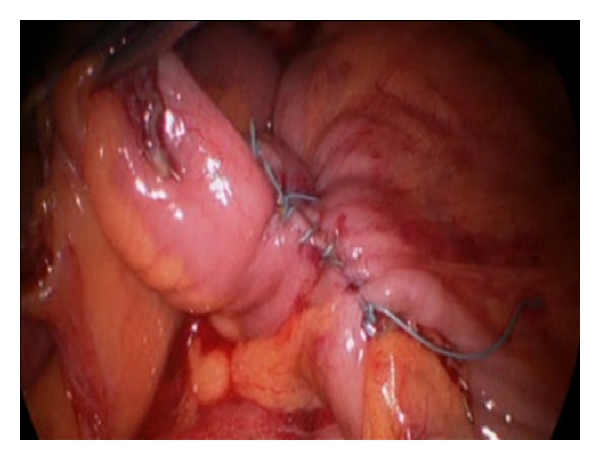
Total intracorporeal anastomosis performed.

The procedure was originally performed through a periumbilical incision, in our first 14 cases, moving into a transumbilical one in the latest 24 cases, what increases patient's satisfaction in term of cosmetic results. No additional trocars were used in any of our cases in order to decrease the trauma of the abdominal wall. We used in all cases a single-port device with two orifices of 5 mm and one of 12 mm (SILS port. Covidien Ltd., Norwalk, CT, USA), a 5 mm 30° scope (Olympus Ltd., Hamburg, Germany), a roticulator grasper (Roticulator Endo Dissect, Covidien Ltd, Norwalk, CT, USA) in the left hand through one of the 5 mm orifice, using the 12 mm orifice to introduce different instruments such as the endoscopic scissors with electrocautery (Roticulator Endo mini-shears, Covidien Ltd., Norwalk, CT, USA), the LigaSure Atlas (Covidien Ltd., Norwalk, CT, USA), originally, while the latest cases has been performed using the LigaSure Advance (Covidien Ltd., Norwalk, CT, USA), the flexible endo-stapler (EndoGIA Roticulator, Covidien Ltd., Norwalk, CT, USA), and the Endo Stitch suture system (Covidien Ltd., Norwalk, CT, USA). Surgery was performed according the standard oncological criteria, following a medial-to-lateral approach with section of ileo-colic vessels close to their origin with the LigaSure (Covidien Ltd., Norwalk, CT, USA). For the exposition of the mesenterium of the right colon, tension was maintained using a suture introduced through the abdominal wall with a straight needle which crossed the abdominal cavity through two distal points between the entry (right lumbar area) and exit sites (suprapubic). This suture was passed through the mesentery close to the ileocecal valve, and it was fixed to the tissue with clips to avoid the suture to slide through the fatty tissue, which allows moving the colon from one side to another by pulling from each side of the suture. This suture allowed the right exposition of the colon during the different phases of the surgery by pulling of the two ends of the suture. Once the main vessels have been divided and the resections of the transverse colon and ileum have been done, a side-to-side intracorporeal anastomosis is performed using a 60 mm Endo Stapler with blue cartridge (Figure 1). The orifice of the anastomosis was closed with a running suture by using the Endo Stitch (Figure 2). The specimen was removed from the abdominal cavity in a 15 mm bag through the same umbilical incision, which was closed with a running absorbable suture under a proper direct vision.

## 3. Results

Twenty-two patients were males (57,9%) and 16 females (42,1%), with an average age of 68,39 years old (range 45–84). Previous clinical history of the patients revealed that 12 of them had previous abdominal surgery. Mean ASA score was 2,71, and the average BMI was 27,88 (range 19,81–41,5).

Lesions were located preoperatively in the cecum in 15 cases (39,5%), in ascending colon in 8 (21,1%), in hepatic flexure in 12 (31,5%), and in transverse colon in 3 (7,9%). Most lesions were adenocarcinoma (25 cases, 65,8%), while the remaining were polyps (12 cases, 31,5%), and one case was due to a previous mucocele of the appendix. Only 17 of these lesions (44,7%) could be detected by the CT scan, while the remaining ones were very small and could not be identified by this imaging technique.

All patients were operated following the same technique, although in 5 of them it was necessary to perform an adhesiolysis due to previous surgery. An extended right hemicolectomy was performed in 17 cases (44,7%), including the transverse colon left to the round ligament, while in the rest of the cases the technique was a standard right colonic resection.

**Figure 3:**
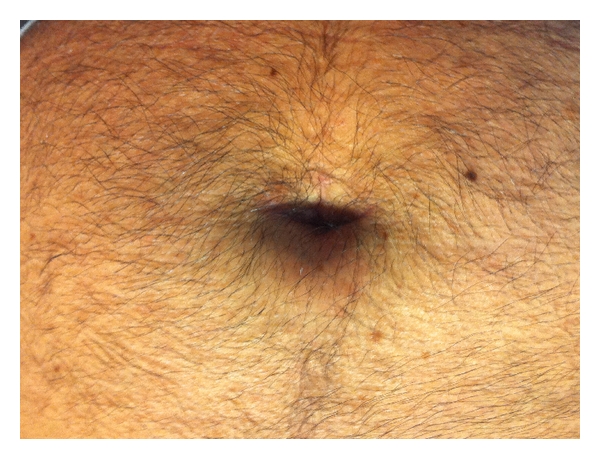
Transumbilical incision one month after surgery.

Regarding the incision, a periumbilical incision was performed in our initial 14 cases (36,8%), while the rest of the cases a transumbilical incision was used (Figure 3). Patient satisfaction increases with the changes in the way that the incision was performed, due to better cosmetic results obtained. Medium size of the incision was 3,25 cm (range 2,5–5,2).

**Figure 4:**
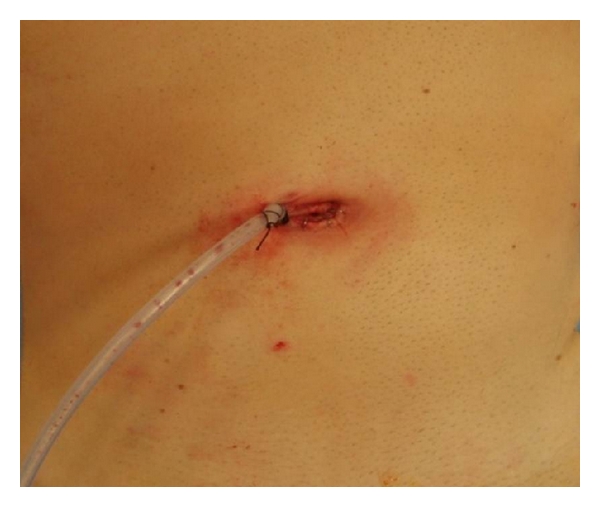
Drain through a periumbilical incision.

Mean surgical time was 117,42 minutes (range 75–190), while the average blood loss during surgery was 118,48 cc. Drain was only used in our first 3 cases, and it was placed through the same periumbilical incision (Figure 4). Drains were not used in the rest of the cases.

Mean hospital stay was 5,2 days, although most of the patients (86,5%) stayed less than 5 days: one patient stayed one day (2,7%), 14 patients 3 days (37,8%), 10 patients 4 days (7%), 7 patients 5 days (19%), 2 patients 6 days (5,4%), and only 3 patients stayed more than 7 days (8,1%).

Regarding complications, we have had one conversion into open surgery, due to a tear of the inferior mesenteric vein. Reoperation rate was 5,2% (2 patients), one due to a bowel obstruction, being performed by conventional laparoscopy, identifying the drain as the cause of this problem, since it entraps the small bowel. The other case was performed by open approach, and it was due to a leak of the anastomosis.

Total morbidity was 13%: there were one leak (2,6%), one bowel occlusion (2,6%), one paralytic ileus (2,6%), and 2 wound infections (5,2%). Long-term follow up showed one incisional hernia (2,6%).

Histological exams of the specimens showed that the oncological criteria, related to number of lymph node (100% patients more of 12 lymph nodes, ranges 12–27) and resection margin (more than 5 cm), were preserved.

## 4. Discussion

We report our initial series of single-port access right hemicolectomy with total intracorporeal anastomosis without any additional trocars. Single-port access surgery is the result of the continuous search for increasing less invasive approaches. This technique has been possible thanks to the development of flexible instruments and trocars which enables the introduction of several instruments [11].

 The main goal of this novel approach is to follow the same steps and principles of standard laparoscopic right hemicolectomy achieving the same oncological results. In fact this laparoscopic approach has been demonstrated to be as effective as conventional surgery for the treatment of carcinoma of colon [1, 2]. Single-port access surgery tries to obtain certain additional benefits in comparison to laparoscopic approach, such as better cosmetic results and potential minimization of postoperative pain, apart from the advantages associated to less traumatism to the abdominal wall, avoiding possible complications associated to the use of additional trocars, such as abdominal wall bleeding or hernias at the site of these additional lateral trocars. But these theoretical advantages still have to be demonstrated in prospective randomized trials.

A review of the literature starts showing different series on single-port right hemicolectomy [12–18]. All series and cases reported were performed with extracorporeal anastomosis, but in our series both the resection of the specimen and subsequent anastomosis were intracorporeal, what could add different advantages to the procedure. In fact, the specimen was removed from the abdominal cavity in a 15 mm bag, avoiding the necessity to enlarge the incision, to carry out the extracorporeal anastomosis, and also possible unnecessary tractions of the pedicle of the transverse colon, where the anastomosis was performed.

Intracorporeal ileocolic anastomosis can be performed safely and effectively, although this technique needs to be performed by expert surgeons with experience in this type of anastomosis and with skills in single-port approach, what could increase the learning curve. On the other hand, this anastomosis could be considered more expensive than the extracorporeal anastomosis, since this last one could be performed manually. Further studies need to analyse if this intracorporeal anastomosis is more cost effective than the extracorporeal ones.

This type of anastomosis has already been described for standard laparoscopic right hemicolectomy in the literature by Bergamaschi et al. [19]. More recently, Bucher et al. [20] have also described an intracorporeal anastomosis in a report of a single-port access gastrojejunostomy, but an additional trocar was added to perform the anastomosis, closing the orifice left by the endostappler with a new special device. However, we defend the use of a running suture to close this orifice, the endostitch being very useful for such purpose as it allows to perform the suture with few wrist movements, avoiding interferences with the scope, since a standard needle holder requires more wrist movements. From a technical point of view, the use of a flexible grasper with the left hand is also important as it allows the exposition of the operation field. However, using straight instruments with the right hand requires a 30° scope to obtain a correct visualization of the tip of them. On the other hand, the suture through the mesentery allows the exposition of the operation field, specially the ileocecal pedicle, replacing standard assistant trocars needed during this procedure.

On the other hand, the use of drain in right colonic resection has been demonstrated not to be necessary, which increases patient satisfaction and decreases postoperative pain. We have moved from the use of drain in our first 3 cases to avoid them. In fact the drain was the cause of one of the reoperations, since it entraps the ileum producing a bowel occlusion. The use of a transumbilical incision, better than a periumbilical one, has increased the cosmetic results of our series.

## 5. Conclusion

Single-port access right hemicolectomy follows the basic principles of conventional right hemicolectomy in term of morbidity and oncological results, although longer followup is necessary to determine the survival. This technique with intracorporeal anastomosis is a safe and feasible approach when performed by experienced laparoscopic surgeons, offering more potential advantages than the extracorporeal anastomosis. The use of transumbilical incision and avoiding additional trocars and drains could increase patient's satisfaction, since it could reduce pain and increase cosmetic results.

Nevertheless, further series and prospective studies must be conducted to prove the effectiveness of this technique in relation to less postoperative pain and less abdominal wall complications while preserving the same oncological results.
